# Optimization Strategies to Adapt Sheep Breeding Programs to Pasture-Based Production Environments: A Simulation Study

**DOI:** 10.3390/ani13223476

**Published:** 2023-11-10

**Authors:** Rebecca Martin, Torsten Pook, Jörn Bennewitz, Markus Schmid

**Affiliations:** 1 Institute of Animal Science, University of Hohenheim, Garbenstr. 17, 70599 Stuttgart, Germany; 2 Animal Breeding and Genetics Group, Department of Animal Sciences, University of Goettingen, Albrecht-Thaer-Weg 3, 37075 Goettingen, Germany; 3 Animal Breeding and Genomics, Wageningen University & Research, P.O. Box 388, 6700 AH Wageningen, The Netherlands

**Keywords:** breeding plans, progeny testing, phenotyping, genotype-by-environment interaction, genetic gain

## Abstract

**Simple Summary:**

Lamb fattening on pasture needs to address consumer demands for the extensive production of animal products with high animal welfare standards and also reduce management load. Therefore, breeding programs should focus on adequate growth and fattening performance in pasture-based production environments. In practice, progeny testing to evaluate fattening performance is commonly performed indoors. This does not represent the actual production environment, which is on pasture in many farms. In this simulation study, different scenarios with varying progeny testing schemes for fattening traits were simulated. The results revealed that a higher number of available phenotypes generally increased the accuracy of breeding value estimation. The highest genetic gain was generated in the scenario with both station and field progeny testing. As the simulation design is highly flexible, the methodology used is applicable in other sheep populations after input data modification. Translating these results into practice could improve the conservation of traditional pasture-based sheep farming in terms of environmental and social factors and increase the income of sheep breeders by improving lamb growth.

**Abstract:**

Strong differences between the selection (indoor fattening) and production environment (pasture fattening) are expected to reduce genetic gain due to possible genotype-by-environment interactions (G × E). To investigate how to adapt a sheep breeding program to a pasture-based production environment, different scenarios were simulated for the German Merino sheep population using the R package Modular Breeding Program Simulator (MoBPS). All relevant selection steps and a multivariate pedigree-based BLUP breeding value estimation were included. The reference scenario included progeny testing at stations to evaluate the fattening performance and carcass traits. It was compared to alternative scenarios varying in the progeny testing scheme for fattening traits (station and/or field). The total merit index (TMI) set pasture-based lamb fattening as a breeding goal, i.e., field fattening traits were weighted. Regarding the TMI, the scenario with progeny testing both in the field and on station led to a significant increase in genetic gain compared with the reference scenario. Regarding fattening traits, genetic gain was significantly increased in the alternative scenarios in which field progeny testing was performed. In the presence of G × E, the study showed that the selection environment should match the production environment (pasture) to avoid losses in genetic gain. As most breeding goals also contain traits not recordable in field testing, the combination of both field and station testing is required to maximize genetic gain.

## 1. Introduction

Most sheep are farmed under extensive production conditions on grasslands, often coping with inadequate nutrition and harsh climate conditions [1,2]. Efficient trait recording is difficult in these environments. Therefore, in breeding programs, traits are often recorded in more intensive production environments or in narrow ranges of environments, e.g., in test stations, and selection is based on these records [3,4]. Due to the discrepancy between the selection environment and the production environment, genotype-by-environment interactions (G × E) might occur. Several studies have demonstrated G × E in sheep [3,5,6,7,8,9].

In Germany, Merino sheep are the predominant breed. In Southern Germany (states of Bavaria and Baden-Wuerttemberg), where nomadic sheep farming is still practiced, this breed accounts for up to 80% of the total sheep population [10]. Lambs are mostly housed together with the ewes and fattened on pasture during the grazing season or, depending on the pasture quality, are fattened on pasture until weaning but subsequently are fattened indoors for finishing. However, there is a trend towards the production of lamb meat reared completely on pasture-based systems because meat from grass-fed lambs is preferred to that from concentrate-fed lambs by the consumers, who associate such systems with better animal welfare [1,11,12]. For lamb meat producers, pasture-based lamb fattening means lower productions costs as lambs are fattened in a low-input system, especially with regard to feeding (e.g., no (or strongly reduced amounts of) concentrate feed). Currently increasing prices for feed concentrates add to the advantages of pasture fattening [13]. Due to its walking and penning ability, the German Merino is additionally well suited for landscape management. To combine the use of sheep in landscape management and profitable lamb meat production in a low input and pasture-based system, adequate growth performance in fattening on pasture is necessary. This is particularly important as it avoids time- and cost-intensive indoor fattening at the end of the fattening period. Within such a system, the breeding goals are adequate fattening performance and carcass quality generated on pasture.

So far, trait recording for Merino lamb fattening traits has mostly been conducted at stations for progeny testing, which has the advantage of being in a highly controllable environment and the capacity to also test hard-to-measure traits like, e.g., feed conversion rate. At station progeny testing for fattening traits (ST), the lambs are fattened indoors with energy- and protein-rich concentrated feed. Thus, ST does not reflect the intended production environment (on pasture). This is expected to cause G × E effects and hence raises the question about expanding trait recording towards field testing (FT) to alleviate these putative G × E effects.

The present simulation study aims to adapt the German Merino sheep breeding program towards pasture-based production systems. A realistic simulation of the current German Merino sheep breeding program was established as a reference to compare with several alternative breeding plans, differing mainly in their progeny testing environment (field and/or station), and assumed a G × E between station and field testing records for fattening traits. The study mainly focuses on fattening performance traits, as the differences in the production environment (indoor fattening vs. fattening on pasture) are expected to be the largest and G × E is likely to occur in German Merino particularly in such traits.

## 2. Material and Methods

### 2.1. Population Simulation and General Breeding Program

**Figure 1:**
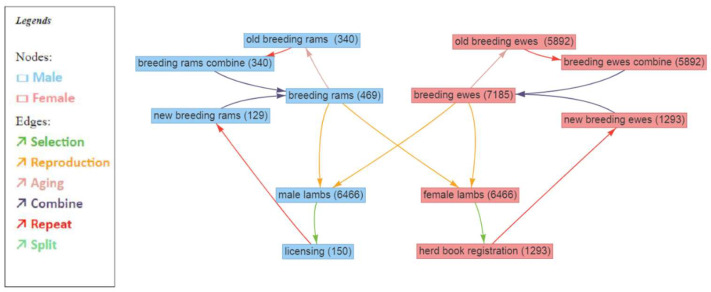
Schematic overview of the simulated German Merino sheep breeding program designed in the user interface of MoBPS (www.mobps.de, [16]). Numbers in brackets indicate the size of the cohort. For more details, see Supplementary File S1.

A simulation of the current breeding program for German Merino sheep was carried out by using the R package Modular Breeding Program Simulator (MoBPS) [14]. The R script of the simulation is provided in Supplementary File S1 and at https://github.com/tpook92/MoBPS, accessed on 9 November 2023. To establish a realistic founder population, real genotype data from 785 German Merino sheep genotyped with an Illumina OvineSNP50 v2 Bead Chip (Illumina, San Diego, CA, USA) were used. Animals were randomly mated for five generations to generate initial relatedness and a pedigree structure. The simulated founder population consisted of 469 breeding rams and 7185 breeding ewes according to the number of herdbook-registered Merino sheep in Bavaria and Baden-Wuerttemberg in January 2022. A general overview of the simulated breeding program is shown in Figure 1. Note that only the herdbook population was simulated because genetic progress was generated only within this population. Thus, all male and female lambs produced were eligible as selection candidates. The transfer of breeding rams to production herds and thus the transfer of genetic gain in these herds was not addressed in this study. In the simulation study, the breeding ram and breeding ewe cohorts were mated to produce male and female lambs with a sex ratio of 1:1. For each mating, an average of 1.8 lambs born per ewe and lambing were assumed. Litter size probabilities were 0.56 for a single lamb, 0.4 for twins and 0.04 for triplets. A probability of 0.1 for lambs dying before the 42nd postnatal day was assumed. These assumptions were based on the latest assessments of the responsible breeding managers (personal communication). The selection steps considered were licensing on the male side (the best 2% of individuals were selected) and herdbook registration on the female side (the best 20% of individuals were selected). Selected males and females replaced those that left the breeding program (i.e., due to culling in real breeding programs). To obtain a realistic age structure with overlapping generations, each individual was simulated to leave the breeding program with a given probability depending on age and sex (see Supplementary Table S5 for more details). This methodology followed that of Büttgen et al. [15] with probabilities of individuals leaving the breeding program based on the observed values in the population of licensed rams in the Bavarian population between 2000 and 2021. The breeding ram cohort was created by combining the breeding rams of the previous breeding cycle that remained in the breeding population for the next breeding cycle (‘old breeding rams’) with new breeding rams selected at licensing (‘new breeding rams’). It was assumed that some males presented at licensing were not licensed and thus were excluded (‘licensing’ to ‘new breeding ram’). The breeding ewe cohort consisted of the breeding ewes of the previous breeding cycle that remained in the breeding population for the next breeding cycle (‘old breeding ewes’) and new breeding ewes selected at herdbook registration (‘new breeding ewes’).

The concept of licensing describes the permission for a ram to be used as a breeding ram. In real breeding programs, herdbook breeders’ preselected males, ideally based on EBVs, are presented to the breeding association’s judges at central licensing and sale events at the age of 15 to 18 months. One requirement for licensing is that the sire of the young ram to be licensed is progeny tested for fattening performance. The licensed ram itself will then be progeny tested after his first lambing. If the licensed ram is sold to a production herd, no further data recording is performed. On the female side, replacement ewes are selected within the herds. Breeders’ preselected females, ideally based on EBVs, are presented to the responsible breeding manager at herdbook registration at an age of 10 to 15 months. Females which meet the requirements are subsequently classified as breeding ewes. Note that in the simulation study, no preselection based on subjective impressions of breeders was simulated.

### 2.2. Trait Simulation

A total of 19 quantitative traits were simulated with 1000 purely additive underlying quantitative trait loci (QTL), which were randomly sampled from the SNP data with effect sizes drawn from a Gaussian distribution (n.additive in *creating.diploid()* in MoBPS [14]. Traits were standardized to a population mean of 100 and a genetic variance of 10. Heritabilities of the traits (Table 1) and genetic correlations between traits were derived from the literature and meaningfully compiled [10,17,18]. A full list of all correlations is given in Supplementary Table S6. To obtain correlated traits, QTL effects of each trait will implicitly also affect other traits (trait.cor in *creating.diploid()* in MoBPS [14]. As the obtained correlation matrix was not positive semidefinite, the matrix was modified by setting all negative eigenvalues of the correlation matrix to zero and thus projecting the matrix in the space of positive semidefinite matrices (see Supplementary File S1 for details). Residual effects between traits were assumed to be uncorrelated. This was assumed because no reliable information was available. The production traits of number of lambs born alive (NL), nursing ability (NA), wool (WL), muscle conformation (MC) and body conformation (BC) were simulated as phenotypes measured on the animal itself (own performance). WL, MC and BC were measured at licensing or herdbook registration, whereas NL was recorded immediately after lambing and NA 42 days post partum. These traits were the only traits measured early in life and were available as phenotypes at the stage of selection. The fattening traits average daily gain (ADG), fleshiness (FLN), ultrasound muscle depth (UMD) and ultrasound fat depth (UFD) were assumed to be measured solely by progeny testing (progeny performance) along with feed conversion ratio (FC), shoulder width (SW), back muscle area (BMA), withers circumference (WC), surface fat area (SFA) and pelvic and kidney fat (PKF). According to the current breeding program, ADG, FLN, UMD and UFD were simulated as fattening traits that could be measured in both FT and in ST. Ultrasound muscle depth and UFD were simulated as proxy traits for carcass performance that are used to indicate whether the lamb is ready to be slaughtered. A trait measured in field testing, FT, is indicated by the subscript _F_, e.g., ADG_F_. A trait measured in station testing, ST, is indicated by the subscript _S_, e.g., ADG_S_. Genetic correlations between the respective traits (rg(ADG_F_, ADG_S_), rg(FLN_F_, FLN_S_), rg(UMD_F_, UMD_S_), rg(UFD_F_, UFD_S_)) were set to 0.8 to simulate G × E between the two progeny testing environments for these four traits (see Supplementary File S1 for details). This level of G × E was chosen as it is frequently discussed as a threshold at which G × E becomes an issue due to re-ranking [19]. According to the current breeding program, the carcass traits (SW, BMA, WC, SFA, PKF) and FC were only assessed in ST. Table 1 provides further information about the traits and their phenotyping timepoints.

### 2.3. Selection of Breeding Animals

Animals were selected according to their total merit index (TMI) based on estimated breeding values (EBVs) and their economic values. Breeding value estimation was conducted for each single trait by applying a BLUP animal model to individuals of the last three generations. For each of the individuals, pedigree information from seven generations was utilized. Breeding values were estimated by using multi-trait BLUP models in the package mixBLUP [20,21]. The genetic parameters were taken from Table 1 and Supplementary File S1. Fixed effects were the trait means and the random effect was the animal effect, with the covariance structure modelled by the pedigree-based relationship matrix. The TMI for an individual was then calculated by weighing the EBVs of the traits. The FT fattening traits (ADG_F_, FLN_F_, UMD_F_ and UFD_F_) were considered in the TMI; the ST fattening traits (ADG_S_, FLN_S_, UMD_S_, UFD_S_) were not included in the TMI but were used to increase the EBV accuracy of the former through their correlation with the FT fattening traits. The TMI weights approximately reflected the currently used TMI weights of German Merino sheep in routine evaluation [22,23]. However, the traits ADG_F_, FLN_F_, UMD_F_ and UFD_F_ are not specifically included there. Since these are of interest for pasture-based breeding programs, these were considered in the present simulation TMI. The TMI was defined by the following index weights and EBVs, with the trait abbreviations explained in Table 1:

TMI = 10 NL + 7.5 WL+ 7.5 MC + 20 BC + 5 NA + 10 ADG_F_ + 2.5 FLN_F_ + 2.5 UMD_F_ + 3.125 UFD_F_ + 15 FC_S_ + 2.5 SW_S_ + 2.5 BMA_S_ + 2.5 WC_S_ + 6.25 SFA_S_ + 3.125 PKF_S_

Breeding value estimation was performed once during each breeding cycle, but selection based on the TMI was performed at two timepoints: First, during the selection of progeny for future breeding animals (transitioning from the male lamb cohort to the licensing cohort or transitioning from the female lamb cohort to the herdbook registration cohort, Figure 1). Second, when sheep remaining in the population for the next breeding cycle were selected (transitioning from the breeding ram cohort to the old breeding ram cohort or transitioning from the breeding ewe cohort to the old breeding ewe cohort, Figure 1). The timepoints of trait phenotyping are shown in Table 1.

### 2.4. Simulated Scenarios

All simulated scenarios followed the general breeding program described above but differed in the progeny testing environment. In all scenarios, individuals were phenotyped for WL, MC and BC. All ewes were phenotyped for NL. The recording status of the trait NA was chosen according to practice and not intended to be optimized. Randomly chosen individuals from the total lamb population (male and female lambs; 25% of the cohort) were phenotyped for NA. Currently, fattening traits in the German Merino sheep breeding program are exclusively measured in station progeny testing. The reference scenario mimicked the currently practiced station progeny testing scheme. Following this, fattening traits were modelled as traits measured exclusively in ST with eight male offspring tested per sire. In the following, the reference scenario is therefore referred to as ST. In total, two alternative scenarios were simulated in which the traits ADG, FLN, UMD and UFD were recorded in FT. Therefore, one scenario was simulated in which only FT was performed (FT) and another in which FT and ST were performed (ST + FT) (see Table 2 for a more detailed scenario description). In FT simulations, the phenotypic records were based on measurements of 20 male lambs per sire. The evaluation of scenarios focused on the TMI, as described earlier. As the traits AGD_F_, FLN_F_, UMD_F_ and UFD_F_ were the central traits of interest, results are additionally presented separately for these traits.

### 2.5. Statistical Analysis

Each scenario was simulated over 20 iterations, i.e., 20 breeding cycles of the breeding program with 50 independent runs of the simulation for each scenario. To generate a realistic age structure, breeding cycles 1 to 9 were excluded from the analysis, and genetic gain was scaled to zero at the starting point in breeding cycle 10. Accordingly, for evaluations of the scenarios, iterations 11 to 20, i.e., 10 full breeding cycles, were considered. For each breeding cycle, the mean genetic gain was calculated as the average of true breeding values, scaled by the mean and genetic standard deviations of the breeding population in breeding cycle 10. Results represent averages across the 50 replicates for the respective scenario. Genetic gain over time was then visualized in plots. The accuracy of breeding value estimation was determined by assessing the correlation of the true breeding values and the EBVs. These metrics were calculated for all scenarios and compared with the reference scenario as well as among scenarios. Paired two-sample Student’s *t* tests were performed to detect significant differences in genetic gain and EBV accuracies between simulated scenarios. *p* < 0.05 indicated significant differences. The results focus on only the males, i.e., the breeding ram cohort and/or the licensing cohort.

## 3. Results

### 3.1. Genetic Gain

**Figure 2:**
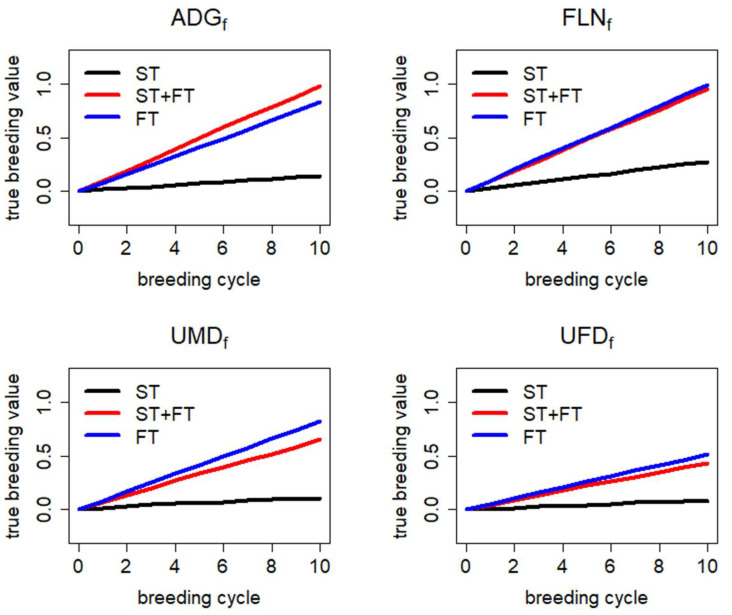
Genetic trend over 10 breeding cycles displayed as the mean true breeding values scaled by genetic standard deviations for fattening traits measured in field progeny testing (FT) [average daily gain (ADG_F_), fleshiness (FLN_F_), ultrasound muscle depth (UMD_F_) and ultrasound fat depth (UFD_F_)]. The reference scenario ST was compared with the alternative scenarios FT and ST + FT. For a detailed trait description, see Table 1. For a detailed scenario description, see Table 2.

For the TMI, the mean true breeding value and standard deviations (SD) per breeding cycle over the 50 independent simulations for each scenario are presented in Table 3 for the breeding ram cohort. Scenario FT showed a slight decrease in genetic gain (−1.0%) compared with the ST scenario in breeding cycle 10, whereas genetic gain in scenario ST + FT (+6.1%) was significantly increased compared with the ST scenario in this breeding cycle. The genetic gain in the single traits ADG_F_, FLN_F_, UMD_F_ and UFD_F_ is shown in Figure 2 (see Supplementary Tables S1–S4 for more details). For all four traits, the genetic gain in the ST scenario was small. Both alternative scenarios (ST + FT and FT) led to a significant increase in genetic gain compared to the reference scenario ST. The differences between the scenarios ST + FT and FT were less pronounced, with significantly higher genetic gain for ADG_F_ in ST + FT, significantly higher genetic gain in UMD_F_ and UFD_F_ in FT and similar genetic gain for FLN_F_. Scenario FT led to substantial reductions in the genetic gain in the fattening and carcass traits measured in ST, with the ST scenario and ST + FT exhibiting similar performance. Supplementary Figure S1 provides an overview of the genetic gain for the fattening and carcass traits measured in station progeny testing.

### 3.2. Accuracy of Breeding Value Estimation

The presented EBV accuracies refer to the average across the 10 breeding cycles and 50 runs in the licensing and breeding ram cohorts. The results are shown in Table 4 for the TMI and the FT traits (ADG_F_, FLN_F_, UMD_F_ and UFD_F_). The EBV accuracy for the breeding ram cohort was generally higher than that for the licensing cohort for the TMI and the four individual traits. Scenario ST + FT led to a significantly higher EBV accuracy compared to the other scenarios for both cohorts. The EBV accuracy in scenario FT was significantly different from the ST scenario in the licensing cohort but not in the breeding ram cohort. For the four FT traits, EBV accuracy in the reference scenario ST was significantly lower than that in both alternative scenarios for all four traits and both cohorts. Accuracies were highest in scenario ST + FT for all four traits and both cohorts. Accuracies in ST + FT were approximately 50% higher for FLN_F_, UMD_F_ and UFD_F_ than that in ST for both cohorts. For ADG_F_, the increase was even more pronounced. The accuracy in scenario FT was significantly lower than that in ST + FT but significantly higher than that in ST for both cohorts and all four FT traits.

### 3.3. Inbreeding and Genetic Variance

The level of inbreeding displayed as kinship increased linearly over the 10 considered breeding cycles but did not exceed a kinship of 0.02 in all scenarios with no significant differences between the scenarios (see Supplementary Figure S2). Regarding the genetic variance, the simulated scenarios had no significant effect (see Supplementary Figure S3).

## 4. Discussion

In pasture-based sheep breeding programs, the breeding goals are lamb growth and fattening performance on pasture. However, traits are often recorded in feed-intensive environments (ST) and thus do not match the actual production environment. Consequently, G × E might occur, which might hamper possible breeding progress. In this simulation study, possible strategies to adapt the German Merino sheep breeding program to the pasture-based production environment were investigated. Alternative scenarios were evaluated that differed in their progeny testing environment (FT, ST, ST + FT).

### 4.1. Study Design

To mimic the existing German Merino sheep breeding program, the software MoBPS [14] was applied. This software allows the flexible simulation of breeding programs due to its modular makeup and the ability to use real population data as input data. Pedigree-based BLUP breeding value estimation was implemented as this practice is currently followed [23]. Selection intensity and generation intervals were not affected by the simulated scenarios and thus were not evaluated during the simulations. The same is valid for the breeding goal, which could also be varied to evaluate the impact of G × E on breeding programs [24]. We focused on the results in the male cohorts. Genetic gain in breeding programs is mostly driven by the sires due to the high selection intensity in this path and the availability of a larger number of phenotypes generated at progeny testing compared with dams. We imposed an influence of the environment on genotypes by assuming a genetic correlation of 0.8 for traits affected by G × E. This correlation implies existing (but not strong) G × E in which a re-ranking of animals is expected with the potential to compromise genetic gain [25]. Lower genetic correlations between a trait measured in different environments are found across countries [19]. The consequences of other levels of G × E on genetic gain could have been investigated by varying the genetic correlation for G × E affected traits. However, this was out of the scope of the study. In the study, G × E was simulated for fattening traits which can be measured in both considered environments (indoors and on pasture) and for which G × E is likely to occur due to the big environmental differences [3]. This requires records of the traits measured in different environments, i.e., indoors and on pasture, which was realized by the use of progeny performance measured in both environments in this study. Because the candidates themselves can only have trait records in one of the environments (no G × E detectable), their own performance was not used as additional information in the breeding program for simplicity, although this would have led to higher EBV accuracies. Such approaches are common in G × E studies [7,8].

### 4.2. Evaluation of the Alternative Scenarios

The alternative scenarios simulated in this study suggest putative optimization strategies to increase genetic gain in a pasture-based production environment. Scenario ST + FT led to significantly higher genetic gain for the TMI compared to both the reference scenario ST and the other alternative scenario FT, as phenotypes were assessed both in FT and ST, thus enabling direct selection on all traits in the TMI. This was also reflected in the EBV accuracy. The reduced EBV accuracy of the licensing cohort compared to the breeding ram cohort was due to a greater utilization of information on closely related animals for the latter. In addition, the breeding ram cohort contained individuals from multiple breeding cycles with greater overall differences and less focus on within-family differences in the estimation. Further, the increased numbers of phenotyped lambs in the alternative scenarios (see Table 2) compared to the reference scenario ST additionally contributed to the increase in EBV accuracy and thus to the higher genetic gain. This was also observed in Grill et al. [26] for fattening traits in Austrian meat sheep. No phenotypes for the FT traits ADG_F_, FLN_F_, UMD_F_ and UFD_F_ were available in the ST scenario. Thus, breeding value estimation was dependent on the ST traits ADG_S_, FLN_S_, UMD_S_ and UFD_S_, which were genetically correlated (0.8) with their FT equivalents. The ST equivalents were, however, not weighted in the TMI, which explains both the low accuracy of EBVs and the low genetic gain in these traits. This was the result intended with the chosen correlation of 0.8. It was expected that the highest genetic gain would occur in scenario ST + FT, as this scenario showed the highest number of phenotyped individuals (eight male lambs in ST plus 20 male lambs in FT) and thus the highest EBV accuracy. However, except for ADG_F_, scenario FT generated the highest genetic gain for the FT traits. This is because for traits that are not recorded, no sufficient genetic gain could be obtained, although they were still present in the TMI. A stronger weighting of the FT traits ADG_F_, FLN_F_, UMD_F_ and UFD_F_ in the TMI would further increase genetic gain, but evaluating changes in the TMI weightings was beyond the scope of the study [26].

### 4.3. Practical Relevance of the Study

Selection index weights need to be derived from economic weights and should include economically relevant traits or traits correlated to economically relevant traits [26,27]. As mentioned above, income is mostly generated by landscape management and lamb meat sales. Good lamb growth and fattening on pasture is essential for combining these two economically important paths to maximize production output and avoid cost-intensive barn fattening at the end of the fattening period in the field (on pasture). In the TMI, weights were also on the carcass traits (SW, BMA, WC, SFA, PKF) and FC measured in ST. Feed conversion is especially difficult to implement in an FT scheme performed on pasture. The intention was to investigate if ST is useful, although fattening on pasture is the breeding goal. The genetic gain results for the TMI support the potential of an FT scheme in addition to ST, as simulated in scenario ST + FT. Another advantage of ST is that heritabilities measured under ST conditions tend to be higher as the environment is more standardized [3].

Studies investigating G × E in fattening traits in German Merino sheep regarding production environments, i.e., indoor fattening and fattening on pasture, were not available. Bishop et al. [3] found significant G × E for live weight in Scottish Blackface lambs reared in an intensive environment (housed with concentrated feed) or in an extensive environment (hill side conditions or improved pasture), thus highlighting the inadequacy of an intensive environment to improve live weight on pasture. Additionally, sheep on pasture suffer from infections with gastrointestinal parasites which have the potential to compromise growth [28]. Lambs fattened indoors, in turn, are not exposed. Consequently, G × E is expected in the German Merino population for fattening traits unless not proofed in real data of the population. Following, G × E was assumed for the four field fattening traits (ADG, FLN, UMD, UFD). These traits have been implemented as traits measured in ST and in FT in the current breeding program; however, field testing is not routinely applied by all breeders. By contrast, no G × E was assumed for the carcass traits (SW, BMA, WC, SFA, PKF) and FC because according to the current breeding program, these traits were implemented only as traits measured in ST due to practical reasons in real breeding programs. In practice, no data recording at slaughtering is performed in FT. This is because only the slaughterhouse of the breeding association records the previously mentioned carcass traits and FC. In commercial slaughterhouses, only the live weight before slaughtering and the carcass weight are recorded [26,29]. According to Mulder and Bijma [25], a trait correlation of 0.8 between the selection environment and the production environment indicates a potential 20% loss in genetic gain if no records on progeny performance in the production environment are obtained. This highlights the need for trait recording in the actual production environment, which is on pasture in many German sheep farms. However, from an individual breeder perspective, ST represents reduced management load. The workload can be outsourced to the institution that performs ST. In contrast, FT means that breeders must weigh sheep on their own. For nomadic shepherds, this imposes a large management load. To obtain ultrasound measurements, employees of the breeding association usually phenotype lambs on farms. From a breeding association perspective, however, implementation costs for FT are less than those for ST. To reduce the management load of breeders but still perform FT, it may be possible to implement an association-based FT of reference rams on pasture, as suggested in Peters [27]. Carcass traits could also be recorded from all lambs raised in FT and not only from individuals tested in ST. This is expected to further increase genetic gain and the accuracy of EBVs. However, field testing for the traits ADG, FLN, UMD and UFD is noninvasive and can be performed with living animals, thus being realistic for routine performance. Grill et al. [30] showed that the ultrasound traits UMD and UFD and ADG are valuable to predict carcass performance in the living animal. This provides the opportunity to phenotype individuals and subsequently select them for breeding. Such an approach might also strongly increase the acceptance of breeders because they are able to keep the best tested females as replacement ewes and can keep or sell the best males as sires. 

Body conformation had the highest TMI weight. This trait is also important in pasture farming. Sheep need to have adequate body conformation and robustness to survive in harsh environments [27]. Body conformation is highly correlated with both MC and WL. Those three traits are typically phenotyped at licensing (herdbook registration) of male (female) lambs. This simulation did not investigate how these traits influence further ADG, but this could be a topic for further investigation. If there is a correlation between conformation traits and fattening traits, unintentional but favorable co-selection on fattening traits could be possible.

### 4.4. Outlook

Montossi et al. [1] and Peters [27] emphasize the need to optimize sheep farming for greater efficiency and reduce resource use but increase lamb meat production output. This is important in times of social (low income from sheep farming, difficulties in hiring young shepherds) and environmental (climate change, feed vs. food debate) limitations [12]. The income of shepherds in Germany is mainly generated by landscape management, with payments generally bound to managed areas. Moreover, consumers demand agricultural products from sustainable production systems with high animal welfare. Therefore, fattening lambs on pasture should be favored. Our results clearly demonstrate opportunities to increase the efficiency of the German Merino sheep breeding program for a pasture-based production environment, if G × E is present, through the implementation of FT. Although this study simulated only the herdbook breeding population, the results are also important for non-herdbook breeders who frequently use sires from the herdbook population in their production herds. Consistent recording of body weights in such production herds enables farmers to select for ADG within these herds on the female side. These records could also be utilized as phenotypic information for sires in breeding programs. Further refinements should be evaluated in future studies to fully address all mechanisms of a breeding program, e.g., evaluating the proportion of lambs in progeny testing schemes, similar to Grill et al. [26], as well as differences caused by selecting animals based on conventional EBVs and different selection strategies based on genomic EBVs, similar to Lillehammer et al. [31].

## 5. Conclusions

The present simulation study used the German Merino sheep breeding population as an example for the identification of alternative strategies for adapting a sheep breeding program to its intended production environment (on pasture). We recommend implementing FT for fattening traits to complement the current ST (scenario ST + FT). Although trait recording is more difficult in pasture environments, this study indicates that efforts will pay off regarding genetic gain in sheep breeding programs for pasture-based production environments.

## Figures and Tables

**Table 1:** Simulated trait names and abbreviations, trait heritabilities (h^2^) and phenotyping timepoints in the simulated German Merino sheep breeding program.

Trait Name	Abbreviation *	Trait h^2^	Phenotyping Timepoint
**Reproduction/Rearing traits**			
Number of lambs born	NL	0.10	Lambing
Nursing ability	NA	0.25	42 days after lambing
Conformation traits			
Wool	WL	0.20	Licensing/Herdbook registration
Muscle conformation	MC	0.25	Licensing/Herdbook registration
Body conformation	BC	0.30	Licensing/Herdbook registration
**Fattening traits**			
Average daily gain (field)	ADG_F_	0.26	FT
Fleshiness (field)	FLN_F_	0.13	FT
Ultrasound muscle depth (field)	UMD_F_	0.22	FT
Ultrasound fat depth (field)	UFD_F_	0.17	FT
Average daily gain (station)	ADG_S_	0.40	ST
Fleshiness (station)	FLN_S_	0.50	ST
Ultrasound muscle depth (station)	UMD_S_	0.26	ST
Ultrasound fat depth (station)	UFD_S_	0.20	ST
**Carcass traits**			
Shoulder width	SW_S_	0.33	ST
Back muscle area	BMA_S_	0.36	ST
Withers circumference	WC_S_	0.14	ST
Surface fat area	SFA_S_	0.12	ST
Pelvic and kidney fat	PKF_S_	0.19	ST
**Efficiency trait**			
Feed conversion ratio	FC_S_	0.40	ST

* The subscript _S_ indicates a trait phenotyped in station progeny testing (ST). The subscript _F_ indicates a trait phenotyped in field progeny testing (FT).

**Table 2:** Description of simulated scenarios.

Scenario Name	Progeny Testing Scheme	Number of Phenotyped Lambs Per Sire	Traits with Available Phenotypes *^1^
ST *^2^	ST	8 male lambs per sire	ADG_S_, FLN_S_, UMD_S_, UFD_S_, SW_S_, BMA_S_, WC_S_, SFA_S_, PKF_S_
ST + FT	ST and FT	8 male lambs per sire (station), 20 male lambs per sire (field)	ADG_F_, FLN_F_, UMD_F_, UFD_F_, ADG_S_, FLN_S_, UMD_S_, UFD_S_, SW_S_, BMA_S_, WC_S_, SFA_S_, PKF_S_
FT	FT	20 male lambs per sire	ADG_F_, FLN_F_, UMD_F_, UFD_F_

*^1^ The subscript _S_ indicates a trait phenotyped in station progeny testing (ST). The subscript _F_ indicates a trait phenotyped in field progeny testing (FT). For a detailed trait description, see Table 1. *^2^ Scenario ST is simulated as the reference scenario.

**Table 3:** Mean true breeding values and standard deviations (SD) across all 50 simulated runs per breeding cycle in breeding cycles 0 to 10 for the total merit index (TMI) for the reference scenario ST (progeny testing on station) and the alternative scenarios ST + FT (progeny testing on station and in the field) and FT (progeny testing in the field) for the breeding ram cohort, and contrasting significances between scenarios.

	ST ^a^		ST + FT ^b^		FT ^a^	
Breeding Cycle	Mean	SD	Mean	SD	Mean	SD
1	0.371	0.033	0.387	0.037	0.364	0.039
2	0.743	0.056	0.769	0.054	0.732	0.056
3	1.116	0.068	1.159	0.069	1.099	0.063
4	1.485	0.076	1.547	0.082	1.458	0.073
5	1.844	0.092	1.940	0.079	1.807	0.083
6	2.213	0.102	2.338	0.089	2.165	0.094
7	2.584	0.109	2.719	0.094	2.524	0.111
8	2.942	0.114	3.096	0.101	2.895	0.114
9	3.285	0.121	3.495	0.11	3.256	0.116
10	3.654	0.133	3.877	0.114	3.616	0.123

^a,b^ Scenarios with different superscripts differ significantly at *p* < 0.05.

**Table 4:** Mean accuracy of estimated breeding values (EBVs) and standard deviations (SD) for the licensing and breeding ram cohorts for the total merit index (TMI) for the fattening traits *^1^ measured in field progeny testing (FT) [average daily gain (ADG_F_), fleshiness (FLN_F_), ultrasound muscle depth (UMD_F_) and ultrasound fat depth (UFD_F_)] among the reference scenario *^2^ ST and the alternative scenarios ST + FT, andFT over 10 breeding cycles.

		Licensing Cohort		Breeding Ram Cohort	
Trait	Scenario	Mean Accuracy	SD	Mean Accuracy	SD
TMI	ST	0.234 ^a^	0.014	0.723 ^a^	0.006
	ST + FT	0.293 ^b^	0.011	0.800 ^b^	0.002
	FT	0.256 ^c^	0.010	0.724 ^a^	0.004
ADG_F_	ST	0.174 ^a^	0.009	0.036 ^a^	0.005
	ST + FT	0.609 ^b^	0.005	0.779 ^b^	0.002
	FT	0.600 ^c^	0.007	0.775 ^c^	0.002
FLN_F_	ST	0.415 ^a^	0.006	0.495 ^a^	0.005
	ST + FT	0.611 ^b^	0.010	0.765 ^b^	0.003
	FT	0.550 ^c^	0.007	0.714 ^c^	0.003
UMD_F_	ST	0.378 ^a^	0.009	0.500 ^a^	0.005
	ST + FT	0.617 ^b^	0.005	0.781 ^b^	0.003
	FT	0.588 ^c^	0.007	0.758 ^c^	0.002
UFD_F_	ST	0.376 ^a^	0.007	0.501 ^a^	0.003
	ST + FT	0.580 ^b^	0.004	0.752 ^b^	0.002
	FT	0.548 ^c^	0.008	0.725 ^c^	0.002

^a–c^ Scenarios with different superscripts differ significantly at *p* < 0.05. *^1^ For a detailed trait description, see Table 1. *^2^ For a detailed scenario description, see Table 2.

## Data Availability

All simulations were run using MoBPS version 1.9.20. The simulation script and software are available at https://github.com/tpook92/MoBPS (accessed on 9 November 2023) and in Supplementary File S1. Genetic data are available from the corresponding author upon request.

## Supplementary Materials

The following supporting information can be downloaded at: https://www.mdpi.com/article/10.3390/ani13223476/s1, File S1: SimulationScript, Table S1: Mean true breeding values and standard deviations (SD) across all 50 simulated runs per breeding cycle in the considered breeding cycles 0 to 10 for the trait average daily gain measured in field progeny testing (ADG_F_) for the reference scenario ST (progeny testing on station) and the alternative scenarios ST + FT (progeny testing on station and in the field) and FT (progeny testing in the field) for the breeding ram cohort and contrast significances between scenarios; Table S2: Mean true breeding values and standard deviations (SD) across all 50 simulated runs per breeding cycle in the considered breeding cycles 0 to 10 for the trait fleshiness measured in field progeny testing (FLN_F_), for the reference scenario ST (progeny testing on station) and the alternative scenarios ST + FT (progeny testing on station and in the field) and FT (progeny testing in the field) for the breeding ram cohort and contrast significances between scenarios, Table S3: Mean true breeding values and standard deviations (SD) across all 50 simulated runs per breeding cycle in the considered breeding cycles 0 to 10 for the trait ultrasound muscle depth measured in in field progeny testing (UMD_F_) for the reference scenario ST (progeny testing on station) and the alternative scenarios ST + FT (progeny testing on station and in the field) and FT (progeny testing in the field) for the breeding ram cohort and contrast significances between scenarios, Table S4: Mean true breeding values and standard deviations (SD) across all 50 simulated runs per breeding cycle in the considered breeding cycles 0 to 10 for the trait ultrasound fat depth measured in field progeny testing (UFD_F_) for the reference scenario ST (progeny testing on station) and the alternative scenarios ST + FT (progeny testing on station and in the field) and FT (progeny testing in the field) for the breeding ram cohort and contrast significances between scenarios, Table S5: Probabilities of individuals (male and female) per age (in months) leaving the breeding program calculated based on the observed values in the population of licensed rams in the Bavarian population between 2000 and 2021 following the methodology of Büttgen et al. [15], Table S6: Simulated genetic correlations (on the lower triangular) between the traits wool (WL), muscle conformation (MC), body conformation (BC), average daily gain in the field (AGD_F_), fleshiness in the field (FLN_F_), ultrasound muscle depth in the field (UMD_F_), ultrasound fat depth in the field (UFD_F_), nursing ability (NA), average daily gain on station (ADG_S_), feed conversion (FC_S_), ultrasound muscle depth on station (UMD_S_), ultrasound fat depth on station (UFD_S_), fleshiness on station (FLN_S_), shoulder width on station (SWS), back muscle area on station (BMA_S_), withers circumference on station (WC_S_), surface fat area on station (SFA_S_) and pelvic and kidney fat (PKF_S_), Figure S1: Genetic trend over the 10 considered breeding cycles displayed as the mean true breeding values scaled by genetic standard deviations for the breeding ram cohort for the single traits recorded at station progeny testing. For average daily gain (ADG_S_), feed conversion ratio (FC_S_), ultrasound muscle depth (UMD_S_), ultrasound fat depth (UFD_S_), fleshiness (FLN_S_), shoulder width (SW_S_), back muscle area (BMA_S_), withers circumference (WC_S_), surface fat area (SFA_S_) and pelvic and kidney fat (PKF_S_), the reference scenario ST (progeny testing on station) was compared with the alternative scenarios ST + FT (progeny testing on station and in the field) and FT (progeny testing in the field), Figure S2: Level of inbreeding over the 10 considered breeding cycles for the breeding ram cohort in the reference scenario ST (progeny testing on station) and the alternative scenarios ST + FT (progeny testing on station and in the field) and FT (progeny testing in the field), Figure S3: Development of genetic variance over the 10 considered breeding cycles relative to the genetic variance in breeding cycle 0 (scaled to 1) for the traits ADG_F_, FLN_F_, UMD_F_ and UFD_F_ for the breeding ram cohort in the reference scenario ST (progeny testing on station) and the alternative scenarios ST + FT (progeny testing on station and in the field) and FT (progeny testing in the field).
