# Supplementary material for: Optimization Strategies to Adapt Sheep Breeding Programs to Pasture-Based Production Environments: A Simulation Study
Source: Animals (Basel). 2023 Nov 10;13(22):3476. doi: 10.3390/ani13223476 (PMC10668732; doi:10.3390/ani13223476)
Supplement: Supplementary file 1 [file animals-13-03476-s001.zip › Supplementary_Tables_Figures.pdf]

Supplementary Materials

Optimization Strategies to Adapt Sheep Breeding Programs to Pasture-Based Production Environments: A Simulation Study

Rebecca Martin [1,*], Torsten Pook [2,3], Jörn Bennewitz [1] and Markus Schmid [1]

[1] Institute of Animal Science, University of Hohenheim, Garbenstr. 17, 70599 Stuttgart, Germany
[2] Animal Breeding and Genetics Group, Department of Animal Sciences, University of Goettingen, Albrecht-Thaer-Weg 3, 37075 Goettingen, Germany
[3] Animal Breeding and Genomics, Wageningen University & Research, P.O. Box 388, 6700 AH Wageningen, The Netherlands
* Correspondence: rebecca.martin@uni-hohenheim.de

Supplementary Table S1

Mean true breeding values and standard deviations (SD) across all 50 simulated runs per breeding cycle in the considered breeding cycles 0 to 10 for the trait average daily gain measured in field progeny testing (ADG_F) for the reference scenario ST (progeny testing on station) and the alternative scenarios ST+FT (progeny testing on station and in the field) and FT (progeny testing in the field) for the breeding ram cohort and contrast significances between scenarios

	Scenario					
	ST[a]		ST+FT[b]		FT[c]	
**Breeding cycle**	**Mean**	**SD**	**Mean**	**SD**	**Mean**	**SD**
1	0.021	0.039	0.101	0.031	0.077	0.035
2	0.033	0.06	0.193	0.057	0.160	0.051
3	0.044	0.072	0.29	0.074	0.244	0.067
4	0.063	0.084	0.393	0.096	0.326	0.083
5	0.075	0.092	0.496	0.116	0.409	0.086
6	0.084	0.103	0.598	0.128	0.487	0.097
7	0.102	0.112	0.693	0.135	0.571	0.106
8	0.117	0.115	0.780	0.146	0.660	0.121
9	0.132	0.121	0.880	0.165	0.745	0.125
10	0.141	0.138	0.981	0.178	0.834	0.133

[a-c] Scenarios with different superscripts differ significantly at $P < 0.05$.

Supplementary Table S2

Mean true breeding values and standard deviations (SD) across all 50 simulated runs per breeding cycle in the considered breeding cycles 0 to 10 for the trait fleshiness measured in field progeny testing (FLN_F), for the reference scenario ST (progeny testing on station) and the alternative scenarios ST+FT (progeny testing on station and in the field) and FT (progeny testing in the field) for the breeding ram cohort and contrast significances between scenarios

	**Scenario**					
	**ST[a]**		**ST+FT[b]**		**FT[b]**	
**Breeding cycle**	**Mean**	**SD**	**Mean**	**SD**	**Mean**	**SD**
1	0.034	0.036	0.093	0.042	0.101	0.038
2	0.063	0.064	0.186	0.063	0.205	0.057
3	0.091	0.071	0.279	0.082	0.307	0.076
4	0.117	0.088	0.383	0.096	0.400	0.093
5	0.140	0.104	0.487	0.113	0.499	0.097
6	0.160	0.123	0.582	0.135	0.591	0.114
7	0.196	0.134	0.664	0.136	0.688	0.124
8	0.227	0.141	0.755	0.148	0.795	0.13
9	0.251	0.151	0.854	0.162	0.892	0.137
10	0.275	0.162	0.951	0.178	0.992	0.151

[a-c] Scenarios with different superscripts differ significantly at *P 151*< 0.05.

Supplementary Table S3

Mean true breeding values and standard deviations (SD) across all 50 simulated runs per breeding cycle in the considered breeding cycles 0 to 10 for the trait ultrasound muscle depth measured in in field progeny testing (UMD_F) for the reference scenario ST (progeny testing on station) and the alternative scenarios ST+FT (progeny testing on station and in the field) and FT (progeny testing in the field) for the breeding ram cohort and contrast significances between scenarios

	**Scenario**					
	**ST**[a]		**ST+FT**[b]		**FT**[c]	
**Breeding cycle**	**Mean**	**SD**	**Mean**	**SD**	**Mean**	**SD**
1	0.013	0.032	0.072	0.043	0.079	0.042
2	0.034	0.053	0.133	0.061	0.167	0.068
3	0.050	0.063	0.199	0.067	0.254	0.096
4	0.060	0.079	0.269	0.083	0.335	0.112
5	0.062	0.091	0.335	0.104	0.416	0.119
6	0.072	0.108	0.397	0.114	0.492	0.137
7	0.087	0.128	0.455	0.122	0.567	0.139
8	0.092	0.128	0.516	0.139	0.66	0.137
9	0.096	0.134	0.581	0.146	0.737	0.152
10	0.107	0.148	0.651	0.156	0.816	0.171

[a-c] Scenarios with different superscripts differ significantly at $P < 0.05$.

Supplementary Table S4

Mean true breeding values and standard deviations (SD) across all 50 simulated runs per breeding cycle in the considered breeding cycles 0 to 10 for the trait ultrasound fat depth measured in field progeny testing (UFD_F) for the reference scenario ST (progeny testing on station) and the alternative scenarios ST+FT (progeny testing on station and in the field) and FT (progeny testing in the field) for the breeding ram cohort and contrast significances between scenarios

	**Scenario**					
	**ST[a]**		**ST+FT[b]**		**FT[c]**	
**Breeding cycle**	**Mean**	**SD**	**Mean**	**SD**	**Mean**	**SD**
1	0.006	0.036	0.043	0.038	0.053	0.04
2	0.016	0.049	0.088	0.059	0.106	0.059
3	0.030	0.068	0.132	0.078	0.157	0.07
4	0.032	0.076	0.178	0.091	0.210	0.086
5	0.038	0.08	0.222	0.104	0.262	0.09
6	0.050	0.094	0.260	0.109	0.311	0.101
7	0.066	0.112	0.298	0.114	0.362	0.113
8	0.072	0.126	0.343	0.124	0.412	0.122
9	0.074	0.13	0.395	0.128	0.463	0.138
10	0.076	0.132	0.434	0.139	0.518	0.147

[a-c] Scenarios with different superscripts differ significantly at $P < 0.05$.

Supplementary Table S5

Probabilities of individuals (male and female) per age (in months) leaving the breeding program calculated based on the observed values in the population of licensed rams in the Bavarian population between 2000 and 2021 following the methodology of Büttgen et al. [15]

Age (in months)	Male	Female
1	0.1208	0.0864
2	0.4155	0.2038
3	0.2633	0.1998
4	0.1256	0.2135
5	0.029	0.1224
6	0.0386	0.0924
7	0.0024	0.045
8	0.0024	0.0217
9	0.0024	0.008
10	0	0.0037
11	0.0024	0.0023
12	1	0
13	-	0.003
14	-	0
15	-	0
16	-	0
17	-	0
18	-	0
19	-	0
20	-	0
21	-	0.0003
22	-	0.0003
23	-	1

Supplementary Table S6

Simulated genetic correlations (on the lower triangular) between the traits wool (WL), muscle conformation (MC), body conformation (BC), average daily gain in the field (AGD_F), fleshiness in the field (FLN_F), ultrasound muscle depth in the field (UMD_F), ultrasound fat depth in the field (UFD_F), nursing ability (NA), average daily gain on station (ADG_S), feed conversion (FC_S), ultrasound muscle depth on station (UMD_S), ultrasound fat depth on station (UFD_S), fleshiness on station (FLN_S), shoulder width on station (SW_S), back muscle area on station (BMA_S), withers circumference on station (WC_S), surface fat area on station (SFA_S) and pelvic and kidney fat (PKF_S)

NL	WL	MC	BC	ADG_F	FLN_F	UMD_F	UFD_F	NA	ADG_S	FCS	UMD_S	UFD_S	FLN_S	SW_S	BMA_S	WC_S	SFA_S	PKF_S
**WL**	1																	
**MC**	0	1																
**BC**	0	0.59	1															
**ADG_F**	0	0.4	0.8	1														
**FLN_F**	0	0	0	0	1													
**UMD_F**	0	0	0	0	0.74	1												
**UFD_F**	0	0	0	0	0.28	0.58	1											
**NA**	0	0	0	0	0	0.29	0.56	1										
**ADG_S**	0	0	0	0	0	0	0	0	1									
**FC_S**	0	0	0	0	0.8	0	0	0	0	1								
**UMD_S**	0	0	0	0	0	0	0	0	0	-0.75	1							
**UFD_S**	0	0	0	0	0	0	0.8	0	0	0.12	-0.01	1						
**FLN_S**	0	0	0	0	0	0	0	0.8	0	0.03	0.02	0.13	1					
**SW_S**	0	0	0	0	0	0.8	0	0	0	0.04	0.29	0.03	-0.01	1				
**BMA_S**	0	0	0	0	0	0	0	0	0	0.1	0.07	0.22	0.02	0.05	1			
**WC_S**	0	0	0	0	0	0	0	0	0	0.15	-0.05	0.38	-0.09	-0.09	0.21	1		
**SFA_S**	0	0	0	0	0	0	0	0	0	0.08	0.02	0.29	-0.09	0.03	0.35	0.53	1	
**PKF_S**	0	0	0	0	0	0	0	0	0	0.05	-0.17	-0.1	-0.15	-0.11	-0.27	-0.02	-0.08	1

Supplementary Figure S1

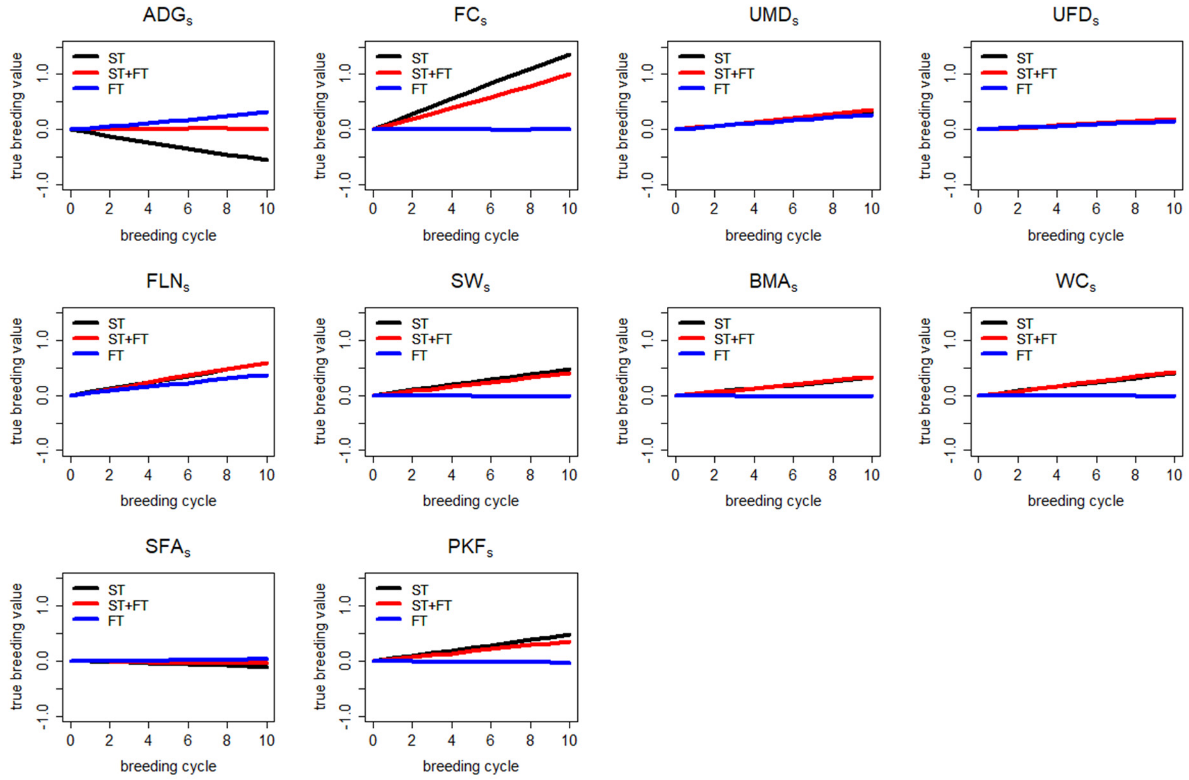

Figure S1: Genetic trend over the 10 considered breeding cycles displayed as the mean true breeding values scaled by genetic standard deviations for the breeding ram cohort for the single traits recorded at station progeny testing. For average daily gain (ADGs), feed conversion ratio (FCs), ultrasound muscle depth (UMDs), ultrasound fat depth (UFDs), fleshiness (FLNs), shoulder width (SWs), back muscle area (BMAs), withers circumference (WCs), surface fat area (SFAs) and pelvic and kidney fat (PKFs), the reference scenario ST (progeny testing on station) was compared with the alternative scenarios ST+FT (progeny testing on station and in the field) and FT (progeny testing in the field)

Supplementary Figure S2

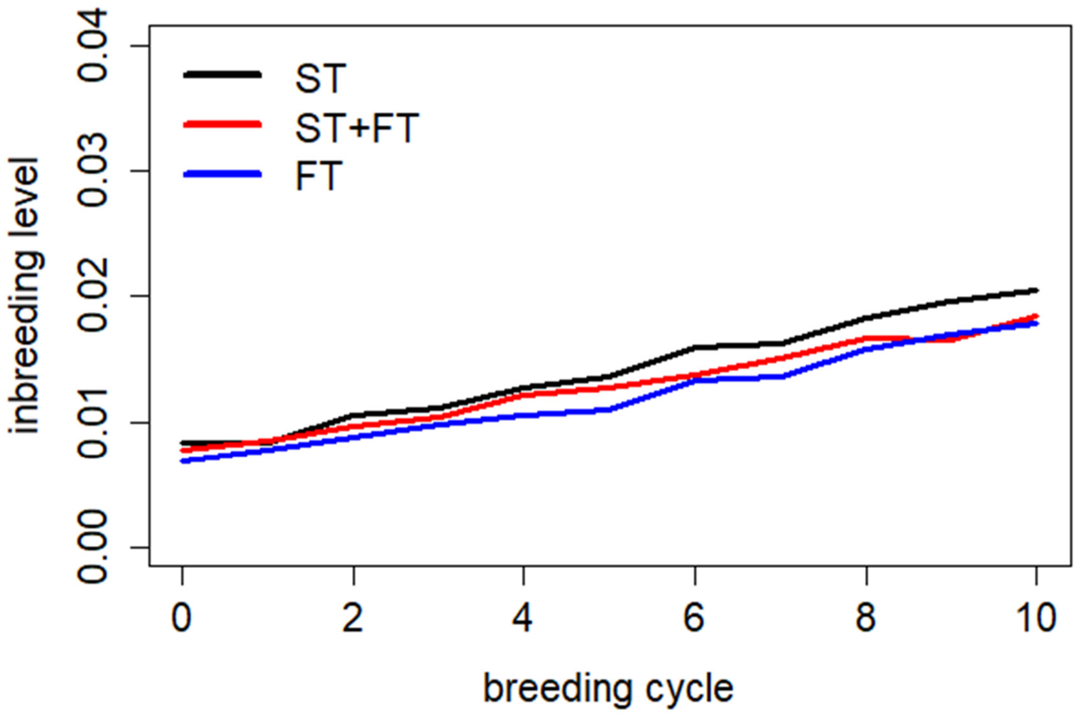

Figure S2: Level of inbreeding over the 10 considered breeding cycles for the breeding ram cohort in the reference scenario ST (progeny testing on station) and the alternative scenarios ST+FT (progeny testing on station and in the field) and FT (progeny testing in the field)

Supplementary Figure S3

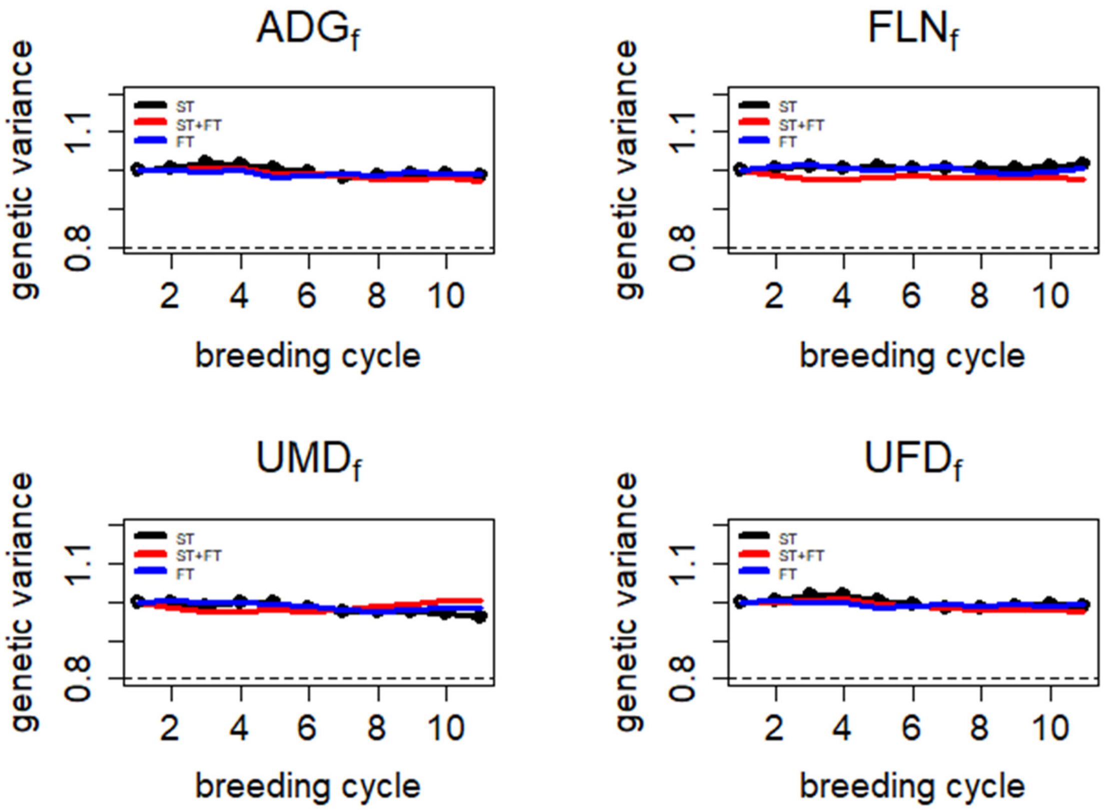

Figure S3: Development of genetic variance over the 10 considered breeding cycles relatively to the genetic variance in breeding cycle 0 (scaled to 1) for the traits ADG_F, FLN_F, UMD_F and UFD_F for the breeding ram cohort in the reference scenario ST (progeny testing on station) and the alternative scenarios ST+FT (progeny testing on station and in the field) and FT (progeny testing in the field)
